# SF_6_^+^: Stabilizing Transient Ions in Helium Nanodroplets

**DOI:** 10.1021/acs.jpclett.1c01024

**Published:** 2021-04-22

**Authors:** Simon Albertini, Stefan Bergmeister, Felix Laimer, Paul Martini, Elisabeth Gruber, Fabio Zappa, Milan Ončák, Paul Scheier, Olof Echt

**Affiliations:** † Institut für Ionenphysik und Angewandte Physik, Universität Innsbruck, A-6020 Innsbruck, Austria; ‡ Management Center Innsbruck, Department Biotechnology & Food Engineering, A-6020 Innsbruck, Austria; § Department of Physics, University of New Hampshire, Durham, New Hampshire 03824, United States

## Abstract

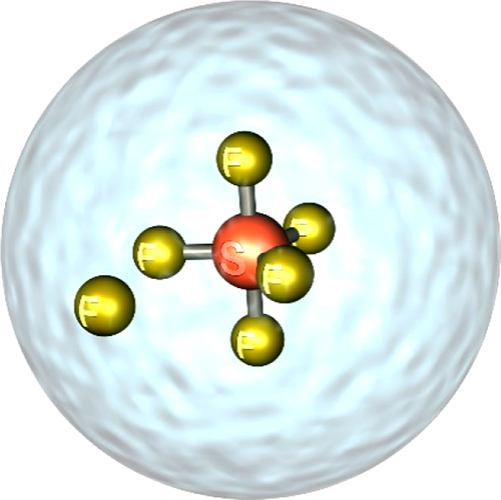

There are myriad ions that are deemed too short-lived to be experimentally accessible. One of them is SF_6_^+^. It has never been observed, although not for lack of trying. We demonstrate that long-lived SF_6_^+^ can be formed by doping charged helium nanodroplets (HNDs) with sulfur hexafluoride; excess helium is then gently stripped from the doped HNDs by collisions with helium gas. The ion is identified by high-resolution mass spectrometry (resolution *m*/Δ*m* = 15000), the close agreement between the expected and observed yield of ions that contain minor sulfur isotopes, and collision-induced dissociation in which mass-selected He_*n*_SF_6_^+^ ions collide with helium gas. Under optimized conditions, the yield of SF_6_^+^ exceeds that of SF_5_^+^. The procedure is versatile and suitable for stabilizing many other transient molecular ions.

SF_6_ is an exceptionally stable molecule, but the SF_6_^+^ cation has never been observed, in spite of numerous attempts involving electron or photon ionization,^1−4^ charge transfer,^5^ photoelectron or photo-ion–photoelectron coincidence spectroscopy,^6,7^ experiments with ultrashort laser pulses,^8^ and isolation in a neon matrix.^9^ Attempts to stabilize SF_6_^+^ in SF_6_ clusters failed, as well.^10−12^ A reduced level of fragmentation upon ionization has been reported for some species embedded in HNDs, but bond rupture cannot be avoided entirely.^13−15^ No SF_6_^+^ ions appear upon ionization of HNDs doped with SF_6_.^16−18^

According to a theoretical study by Bauschlicher and Ricca at the CCSD(T) level of theory, SF_6_^+^ consists of a slightly distorted SF_5_^+^ and a F atom whose dissociation energy is 62 meV.^19^ Failure to produce long-lived SF_6_^+^ is due to the large (1.2 eV) difference between the vertical and adiabatic ionization energies of SF_6_.^6,20^ Kinetic energy release measurements show that ionization leads to impulsive dissociation.^4^ According to an ab initio dynamics study, vertical ionization of isolated SF_6_ produces F + SF_5_^+^ fragments that will separate within 0.2 ps with ∼1.1 eV channeled into kinetic energy.^21^ The challenge to the experimentalist is to quench this fierce reaction.

The mass spectra displayed in Figure 1 demonstrate that this can be done by doping preionized HNDs with SF_6_ in a pick-up cell and then gently stripping the helium matrix in a separate evaporation cell that is filled with low-density helium gas (pressure *P*_evap_) until bare SF_6_^+^ or SF_6_^+^ complexed with a small number of helium atoms emerges.

**Figure 1:**
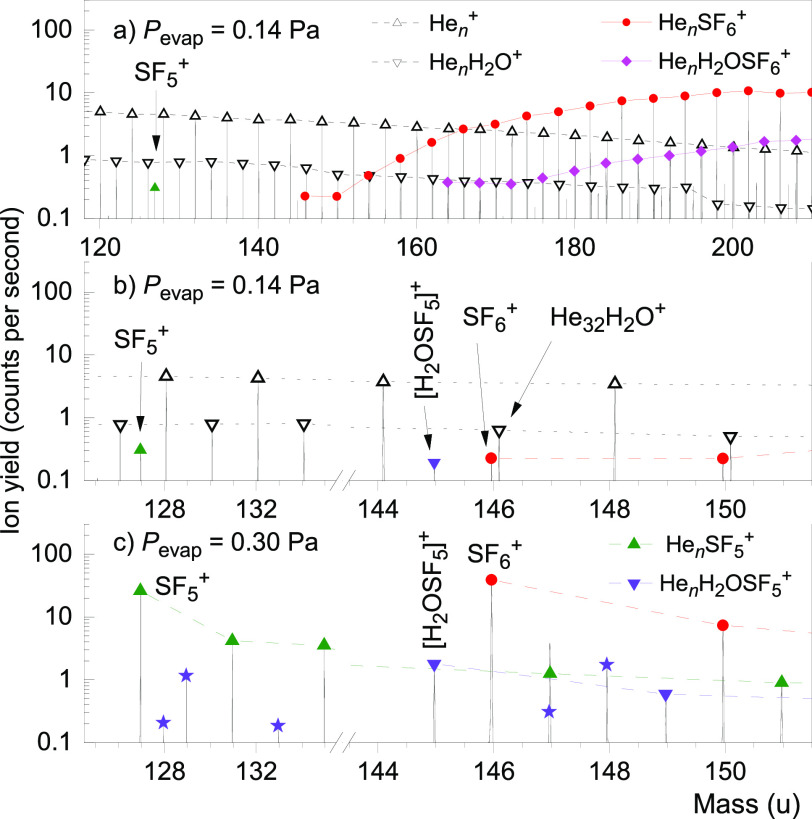
(a) Mass spectrum of charged HNDs doped with SF_6_ recorded at a low evaporation pressure (*P*_evap_ = 0.14 Pa). (b) Expanded view of the spectrum shown in panel a. (c) Mass spectrum recorded with a *P*_evap_ of 0.30 Pa. Prominent ion series are labeled. Expected contributions from ions containing the minor isotopes ^33^S or ^34^S are marked with asterisks; they agree with observations except for the peak at 147 u in panel c that has contributions from [H^32^SF_6_]^+^.

The spectrum in Figure 1a was recorded with a relatively low pressure *P*_evap_ of 0.14 Pa. The ion series He_*n*_^+^ and He_*n*_H_2_O^+^ (*n* ≥ 0) dominate below ≈160 u; their mass peaks are connected by lines. Two singular mass peaks at 127 and 145 u are due to SF_5_^+^ and [H_2_OSF_5_]^+^, respectively (fluorine, mass of 19 u, is monoisotopic; the main isotope of sulfur is ^32^S, with a natural abundance of 94.93%^22^). Two other ion series commence at 146 and 164 u; they are assigned to He_*n*_SF_6_^+^ and He_*n*_[H_2_OSF_6_]^+^, respectively.

Figure 1b presents an expanded view of the top spectrum. Mass peaks assigned to SF_6_^+^ and HeSF_6_^+^ are easily resolved from mass peaks due to He_32_H_2_O^+^ and He_33_H_2_O^+^, respectively, which have the same nominal mass. Possible contributions from another ion at 146 u, [SF_5_H_3_O]^+^, can be safely excluded. It is calculated to be stable, but its mass does not match the spectrum (see the Supporting Information for details).

When the helium evaporation pressure is increased to 0.30 Pa (Figure 1c), the He_*n*_^+^ and He_*n*_H_2_O^+^ ion series disappear; the observed ions can be attributed to He_*n*_SF_5_^+^, He_*n*_[H_2_OSF_5_]^+^, and He_*n*_SF_6_^+^. SF_6_^+^ forms the largest peak. Some mass peaks are due to ions containing ^33^S or ^34^S (natural abundance of 0.76% or 4.29%, respectively). Their expected yields, indicated by asterisks, match the observed yields, except for the peak at 147 u that has contributions from He_5_^32^SF_5_^+^, ^33^SF_6_^+^, and H^32^SF_6_^+^ that has been observed previously.^23^

Many other mass spectra were recorded, with the evaporation pressure ranging from 0.12 to 0.97 Pa. Figure 2 displays the pressure dependence of the ion signal of SF_6_^+^, SF_5_^+^, and He_*n*_SF_6_^+^, the latter summed over 1 ≤ *n* ≤ 200. Between 0.20 and 0.30 Pa, the SF_6_^+^ signal exceeds the SF_5_^+^ signal. Furthermore, the signal of SF_6_^+^ reaches a maximum while that of SF_5_^+^ is still increasing. These observations suggest that SF_6_^+^ is an intermediate dissociation product
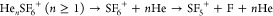
1

**Figure 2:**
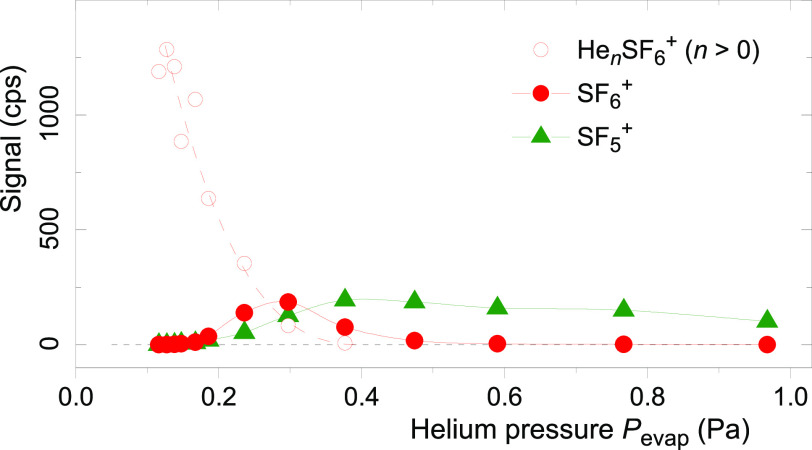
Yield of He_*n*_SF_6_^+^ (summed from *n* = 1–200), SF_6_^+^, and SF_5_^+^ ions vs pressure *P*_evap_ in the evaporation cell. Lines are drawn to guide the eye.

We have tested the hypothesis by colliding mass-selected He_*n*_SF_6_^+^ with low-density helium gas in a collision cell located past the evaporation cell. Two representative mass spectra of the products of CID of He_8_^32^SF_6_^+^ at an energy *E*_lab_ of 20 eV (in the lab frame of reference) are displayed in panels a and b of Figure 3. In panel a, no gas was introduced into the collision cell; 93% of all precursor ions survive the transit through the cell. The value implies a lower limit of 1.0 ms for the lifetime of He_8_SF_6_^+^ (see the Supporting Information). In panel b, the collision gas pressure (*P*_coll_) was increased to 2.0 mPa. Most of the precursor ions dissociate into SF_6_^+^, SF_5_^+^, and some He_*n*_SF_6_^+^ (0 < *n* < 8). He_*n*_SF_5_^+^ (0 < *n*) is virtually absent, thus proving that reaction 1 dominates over the alternative reaction that would bypass formation of SF_6_^+^
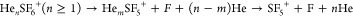
2

**Figure 3:**
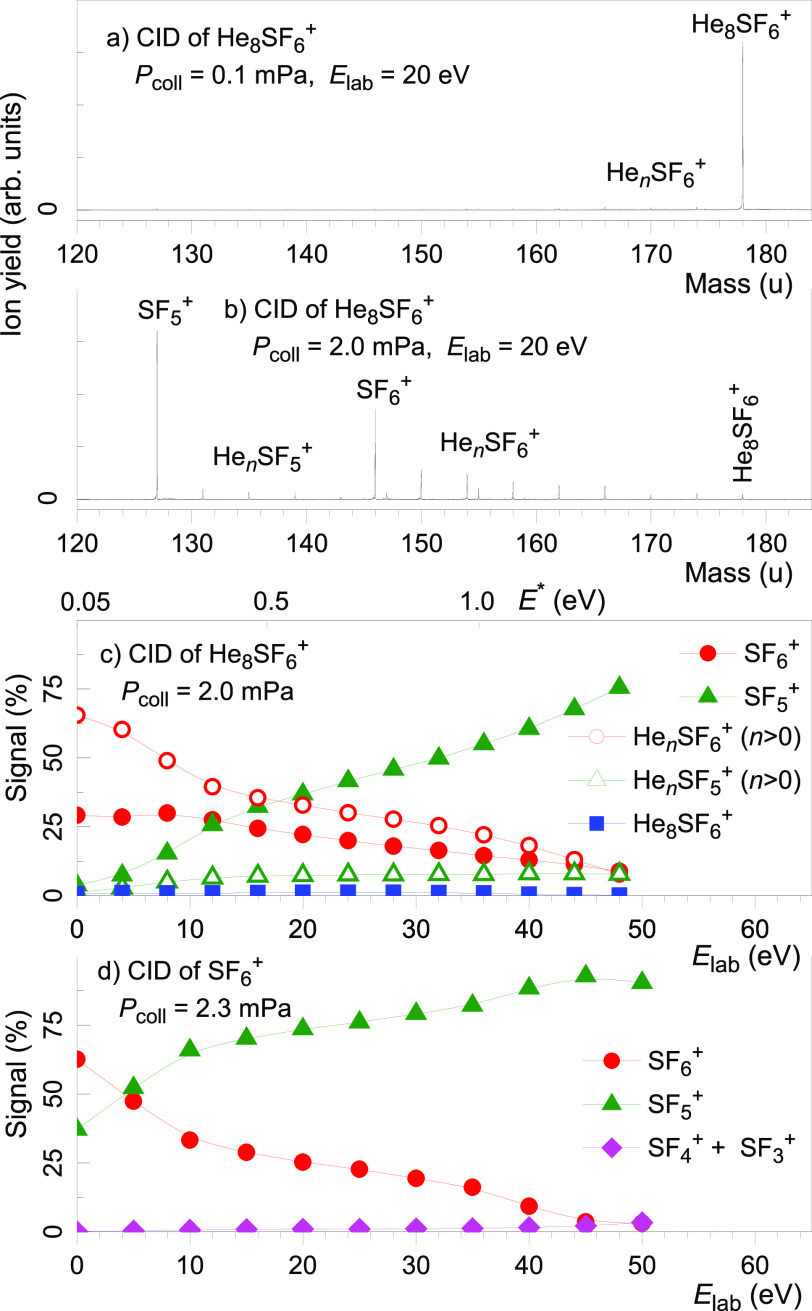
(a and b) Collision-induced dissociation of He_8_^32^SF_6_^+^ at a lab energy *E*_lab_ of 20 eV, at collision gas pressures (*P*_coll_) of 0 and 2.0 mPa, respectively. The spectrum in panel a was recorded in the absence of a collision gas, at a background pressure of 0.1 mPa. Panels c and d display the relative ion yields vs *E*_lab_ for He_8_^32^SF_6_^+^ and ^32^SF_6_^+^ precursor ions, respectively.

The energy dependence of the CID fragment ion yield from He_8_SF_6_^+^ versus collision energy is displayed in Figure 3c for a *P*_coll_ of 2.0 mPa. The signal for He_*n*_SF_6_^+^ represents the sum over 0 < *n* < 8; the total ion yield is normalized to 100%. At the lowest energy, approximately one-third of the ions have fragmented into SF_6_^+^, two-thirds are of the form He_*n*_SF_6_^+^, but barely any ions have dissociated into He_*n*_SF_5_^+^ (0 ≤ *n*). Again, this demonstrates that reaction 1 prevails over reaction 2. He_*n*_SF_6_^+^ sheds all of its helium atoms before it loses a fluorine. Qualitatively similar results were obtained for CID of He_4_SF_6_^+^.

Note that the ion yield of the precursor He_8_SF_6_^+^ in Figure 3c is close to zero, even when *E*_lab_ = 0. Why? The initial speed of the HNDs and, more importantly, the thermal motion of the collision gas provide an offset of ∼0.05 eV to the average energy transfer E* per collision^24,25^ that exceeds the dissociation energy of the parent ion (see below). Values of E* are indicated along the upper abscissa in Figure 3c.

CID data of mass-selected SF_6_^+^ are compiled in Figure 3d. SF_5_^+^ is the dominant fragment ion. Small amounts of SF_4_^+^ and SF_3_^+^ appear at high collision energies; no other fragment ions are detected. This observation further strengthens our conclusion that the mass peak at 146 u is due to ^32^SF_6_^+^. First, the mass of the peak assigned to SF_6_^+^ in Figure 1 agrees with the expected mass within better than 0.0005 u (see the Supporting Information). Second, the observed yield of ions containing the minor sulfur isotopes ^33^S and ^34^S matches the predicted one. Third, the pattern of CID products of ions assumed to be He_*n*_SF_6_^+^ (*n* = 0, 4, or 8) is consistent with the assignment, namely loss of neutrals with a mass of either 4 or 19 u.

The prevalence of reaction 1 over reaction 2 suggests that He is less strongly bound to SF_6_^+^ than F to SF_5_^+^ (assuming that there are no reverse energy barriers for loss of He or F for these reactions). SF_6_^+^ is often assumed to be unstable in its electronic ground state,^1,9^ but some authors have acknowledged that F-SF_5_^+^ may form a weakly bonded complex.^19,26−28^ We considered two isomers of the SF_6_^+^ complex (see Figure 4 and the Supporting Information): (a) isomer **Ia** in which the F atom is dissociated from the structure of the SF_6_ neutral molecule of *O*_*h*_ symmetry, with the symmetry reduced from *C*_4*v*_ to *C_s_*, the F atom being distorted from its original position along the linear F–S–F axis, moving from the equatorial plane of SF_6_, and (b) isomer **Ib** with a F atom attached to SF_5_^+^ in the *D*_3*h*_ bipyramidal structure as a prolongation of a S–F axis, lying 44 meV higher than **Ia**. The calculated dissociation energy of the F atom from **Ia** of 87 meV and the S–F distance of 3.21 Å are close to the previously calculated values;^19^ the difference can be most probably traced to optimization on the B3LYP level in the respective publication. HeSF_6_^+^ complexes were then modeled by adding a helium atom to different sites of the **Ia** isomer, leading to three different structures (**IIa–IIc**). In the most stable **IIa** structure, the helium atom lies among three S atoms, on the other side with respect to the weakly bound sulfur atom.

**Figure 4:**
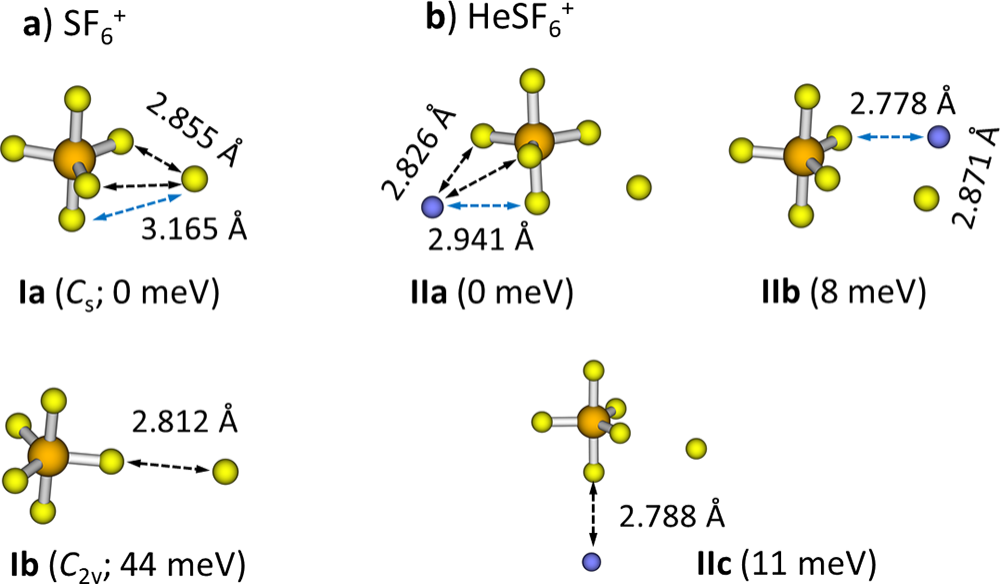
(a) Two isomers of the SF_6_^+^ complex. (b) Three isomers of He (blue) bound to SF_6_^+^. Relative energies are given, optimized at the CCSD/aug-cc-pVDZ level of theory with single-point recalculation at the CCSD(T)/aug-cc-pVTZ level.

To model dissociation of the HeSF_6_^+^ complex (see the Supporting Information), we calculated reaction energies along two different decomposition channels:
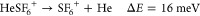
3a
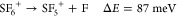
3b
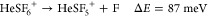
4a
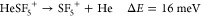
4b

The disparity between the energies for reactions 3a and 4a readily explains the low yield of He_*n*_SF_5_^+^ (*n* > 0) among the collision products of He_8_SF_6_^+^ (Figure 3). The position of the helium atom with respect to SF_6_^+^ plays a rather limited role in energetic considerations (Figure 4). These results are consistent with the experimental observation that, under suitable conditions, SF_6_^+^ is the preferred product ion of He_8_SF_6_^+^ and He_4_SF_6_^+^. Furthermore, they suggest that our experimental approach may be used to stabilize any transient molecular ion AB^±^ provided its binding energy A-B^±^ exceeds its binding energy with helium. Species of potential interest include stable compounds of the light noble gases,^29^ ions whose absorption spectra have so far been measured only in neon or argon matrices,^2^ or labile organic ions that are important intermediates in atmospheric extraterrestrial chemistry.^30,31^

The observation of long-lived SF_6_^+^ contrasts with previous failures to form these ions by doping neutral HNDs followed by ionization.^16−18^ It is usually a thankless task to explain past failures, but we believe that we can rationalize the different outcomes.

We start by outlining the likely sequence of events when a charged HND picks up an SF_6_. In an undoped HND, the charge will be localized on He_2_^+^ or He_3_^+^.^32^ Upon pickup of SF_6_, the dopant and the charge will move toward each other through the superfluid medium; charge transfer will release ∼8.0 eV (see the Supporting Information). Given the large (1.1 eV) energy that is channeled into the F + SF_5_^+^ fragments upon vertical ionization,^21^ and the disparity in the mass of F and He, whether the helium matrix can fully cage the products is questionable. However, all that is needed to stabilize SF_6_^+^ is that the fluorine atom is, with some reasonable probability, thermalized and trapped in the HND. The separate F + SF_5_^+^ pair can then recombine later, even if a solidlike snowball surrounding SF_5_^+^^33−35^ may delay recombination until most of the helium has been stripped in the evaporation cell.

If the scenario described above applies to charged HNDs, why should not it apply to neutral, doped HNDs that are subsequently ionized? In this situation, the dopant will be ionized either via charge transfer from He_2_^+^, which releases 8 eV, or from the primary He^+^, which releases 10.5 eV (see the Supporting Information). In relative terms, these energies are not that much different.

What, then, causes the difference in observations in experiments with charged and neutral HNDs? The difference is primarily one of perception. There are basically two outcomes when a dopant embedded in a large HND containing *N* He atoms is ionized: ion ejection and ion trapping. Ejection leads to small ions He_*n*_X^+^ where *n* ≪ *N*; trapping leads to large ions He_*N–n*_X^+^ (X is the dopant or a fragment thereof). The two outcomes have been nicely illustrated in a recent mixed quantum-classical dynamics simulation of He_1000_Ar_4_ by Halberstadt and Bonhommeau.^36^ Experiments on undoped HNDs (where a tightly bound He_2_^+^ takes on the role of the dopant) also demonstrate the coexistence of these two channels, although in this situation Coulomb repulsion between multiple charges also plays a role (see the Supporting Information and ref (37)). The relative yields of the two competing channels will depend on the nature of the dopant, the size *N*, and other factors, but coexist they will.

Conventional experimental setups are blind to ions trapped in large HNDs because the mass of He_*N–n*_X^+^ exceeds the range of most mass spectrometers; they will detect only ejected ions. Ejection is violent; fragile ions such as SF_6_^+^ are unlikely to survive.^13,14,38^ Conversely, our current approach is blind to small ejected ions because ejection will already happen in the pickup cell; those ions are not guided by electric fields to the mass spectrometer. Only trapped ions will stay on the beam axis. Multiple collisions in the subsequent evaporation cell are needed to gently extract the trapped ions from the HND, unveiling the hitherto unobservable, fragile SF_6_^+^.

In conclusion, we have formed long-lived SF_6_^+^, previously believed to be a transient ion. The experimental approach, doping ionized HNDs and subsequently stripping excess helium by gentle collisions with helium gas, is generic; many other so-called transient ions can probably be stabilized similarly. The only obvious prerequisite, namely that the binding energy of A-B^±^ exceeds that of He-AB^±^, should be met for most systems. The long-range interaction between A and B^±^ is the ion-induced dipole interaction that is particularly weak for He-AB^±^ because of the low polarizability of helium.

Experimental and computational methods: SF_6_^+^ ions are formed in and gently extracted from HNDs as follows. Neutral HNDs are grown by supersonic expansion of helium through a nozzle (diameter of 5 μm, temperature of 9.8 K, and stagnation pressure of 28 bar) into ultrahigh vacuum. The expanding beam is skimmed and ionized by electrons (energy of 37.7 eV and current of 473 μA). The resulting He_*N*_^*z*+^ ions are weakly accelerated into an electrostatic hemispherical deflector set to transmit HNDs with a size-to-charge ratio (*N*/*z*) of ≈3.5 × 10^5^. For the sake of simplicity, we discuss ions that are singly charged. The fate of multiply charged HNDs is discussed in the Supporting Information and elsewhere.^39^

The charged HNDs pass through a pickup cell containing SF_6_ gas at ∼250 μPa, and an “evaporation cell” that contains helium at low, variable pressure *P*_evap_. Each collision will transfer, on average, 0.05 eV to the HND, ∼80 times the evaporation energy of bulk helium.^24,25^ Multiple collisions will lead to partial or complete evaporation of helium from the doped HND. The ions are guided by a radiofrequency field into the extraction region of a time-of-flight mass spectrometer (TOFMS) equipped with a reflectron in W configuration.

Dissociation channels are determined by passing the ions that emerge from the evaporation cell through a quadrupole mass filter. The selected precursor ions are accelerated and sent into a cell where they collide with a thermal gas of helium; product ions are analyzed in the TOFMS. Additional experimental details have been published elsewhere.^24,39,40^

We have calculated SF_5_^+^, SF_6_^+^, their complexes with a single helium atom, and [SF_5_H_3_O]^+^ using the coupled cluster theory (CC), optimized at the CCSD/aug-cc-pVDZ level of theory with single-point recalculation at the CCSD(T)/aug-cc-pVTZ level, including zero-point correction at the CCSD/aug-cc-pVDZ level; calculations were performed in the Gaussian software.^41^ “Very tight” optimization criteria were used for complexes with helium; wave function stability was tested for each structure.

## Supporting Information Available

Fate of multiply charged HNDs, the lifetime of He_8_SF_6_^+^, energy release upon charge transfer to SF_6_, mass analysis, and theoretical results for SF_6_^+^, HeSF_6_^+^, and [SF_5_H_3_O]^+^ (PDF)
