# Supplementary material for: SF6+: Stabilizing Transient Ions in Helium Nanodroplets
Source: J Phys Chem Lett. 2021 Apr 22;12(17):4112–7. doi: 10.1021/acs.jpclett.1c01024 (PMC8154854; doi:10.1021/acs.jpclett.1c01024)
Supplement: Supplementary file 1 — jz1c01024_si_001.pdf [file jz1c01024_si_001.pdf]

Supporting Information

SF_6^+: Stabilizing Transient Ions in Helium Nanodroplets

Simon Albertini, Stefan Bergmeister, Felix Laimer, Paul Martini, Elisabeth Gruber, Fabio Zappa, Milan Ončák,* Paul Scheier,* Olof Echt*

* E-mail addresses: milan.oncak@uibk.ac.at, paul.scheier@uibk.ac.at, olof.echt@unh.edu

S1. The Fate of Multiply Charged HNDs in the Pickup and Evaporation Cells
S2. The Lifetime of $He_8SF_6^+$
S3. The Energy Released upon Charge Transfer to SF_6 Embedded in a HND
S4. Mass Analysis
S5. Theoretical Results for SF_6^+ and $HeSF_6^+$
S6. Theoretical Results for $[SF_5H_3O]^+$
S7. References

S1. The Fate of Multiply Charged HNDs in the Pickup and Evaporation Cells

Multiply charged He_N^{z+} droplets become unstable with respect to spontaneous fission when their size N falls below the critical size $N_z = 3.54\times10^4 z^{3/2}$, i.e. $N_2 = 10^5$, $N_3 = 1.84\times10^5$, etc.[1] These values have been measured for undoped HNDs but there is no reason why they should not also apply to doped HNDs as long as the volume occupied by the dopant is small. In the present work, HNDs with $N/z \approx 3.5\times10^5$ were selected; their maximum possible charge state is $z_{max} = 97$. A more realistic upper limit is $z_{\underline{max}} = 26$, computed from the average size of the neutral HNDs, $N_{av} \approx 4\times10^6$. The distribution of z will also depend on the energy and current of the ionizing electrons. The charges will be localized on He_2^+ (or He_3^+, see S3 below) which will reside near the surface of the HND because of the mutual Coulomb repulsion.

When a z-fold charged HND picks up a SF_6, charge transfer from one of the embedded He_2^+ to SF_6 will release approximately 8 eV (see S3 below) which exceeds the cohesive energy of bulk helium by a factor 1.3×10^4. If the ion remains trapped in the HND, the energy will be dissipated by evaporation of that many helium atoms. The new size N may drop below the critical size of the current charge state, and the HND will fission. A very small singly charged dopant ion dressed with a few He atoms will be ejected; the remaining z-1 charges will remain in the HND that retains nearly all the He atoms.[1] The light fragment will recoil out of the beam; the heavy one will remain near the beam axis.

Another SF_6 colliding with the HND in the pickup cell may come close to another embedded He_2^+ releasing, once again, about 8 eV upon charge transfer, or it may attach to a SF_6^+ (or SF_5^+) and release a much smaller energy. Either way, the droplet will keep shrinking.

Eventually a droplet of reduced size and charge will exit the pickup cell and enter the collision cell where it suffers multiple collisions with thermal helium gas. Each collision transfers about 50 meV to the HND,[2] causing the evaporation of about 80 He atoms. At some point the size of the doped HND drops below 5×10^4, the critical size of doubly charged droplets, and it fissions one last time. After another ≈600 collisions the doped singly charged droplet will have shed all or nearly all of its He atoms, and it will appear in the mass region of interest.

S2. The Lifetime of $He_8SF_6^+$

SF_6^+ ions that are observed must have survived for about 3 ms, from the exit of the evaporation cell to the extraction region of the mass spectrometer. Their mean lifetime can be obtained from the CID measurements. When mass-selected $He_8SF_6^+$ ions pass through the collision cell (which is not deliberately filled with a collision gas), the ion beam exiting the cell consists of $He_8SF_6^+$ (92.6 %) and smaller $He_nSF_6^+$ (7.4 %, summed over $n < 8$). Other ions are absent (see the mass spectrum in Fig. 3a).

The distance between the exit of the quadrupole mass filter and the region where ions are extracted into the TOFMS measures 0.37 m. If we assume that the precursor ions do not suffer any collisions in the collision cell, the lifetime τ of the ions is obtained from $I = I_0 \exp(-t/\tau)$ with the transit time $t = 79.5$ μs calculated from the ion energy (E_{lab} = 20 eV). One obtains $\tau = 1.03$ ms. This represents a lower limit to the true lifetime because the pressure in the collision cell is finite even if no gas is introduced.

S3. The Energy Released upon Charge Transfer to SF_6 Embedded in a HND

The adiabatic ionization energies (AIE) of He, He_2 and He_3 can be derived from the IE of He (24.59 eV) and the adiabatic dissociation energies of He_2^+ (2.35 eV) and He-He_2^+ (0.17 eV).[3,4] Thus, the adiabatic IEs of He_2^+ and He_3^+ equal 22.24 eV and 22.07 eV, respectively. The AIE of SF_6 equals 14.11 eV.[5]

If charge transfer between SF_6 and He^+, He_2^+ or He_3^+ were to occur in the gas phase, an energy of 10.5 eV, 8.1 eV, or 8.0 eV, respectively, would be released. Charge transfer within a helium droplet will change these values slightly because of the polarization of the surrounding helium.

S4. Mass Analysis

The mass resolution equals about 15 000, measured at full-width-at-half-maximum (FWHM), corresponding to a FWHM = 0.010 u at 146 u. The position of a mass peak, obtained by fitting a Gaussian, is determined with a precision of ±0.0003 u. The presence of He_n^+ ions provides a highly accurate mass calibration. If the mass peaks due to He_{36}^+ (144 u) and He_{37}^+ (148 u) are used to calibrate the mass scale, the mass of the two peaks observed at 146 u agrees with the values expected for $^{32}SF_6^+$ and $He_{32}H_2O^+$ within better than 0.0005 u, see Fig. S1.

One might argue that the mass peak at 146 u is due to a hypothetical $[SF_5H_3O]^+$ rather than SF_6^+. However, the mass of the former exceeds that of the latter by 0.020 u, about twice the full-width-at-half-maximum in this mass range. The mass spectrum in Fig. S1 illustrates that the presence of $[SF_5H_3O]^+$ can be safely ruled out. The calculated geometry and energetics of $[SF_5H_3O]^+$ are presented in Section S6.

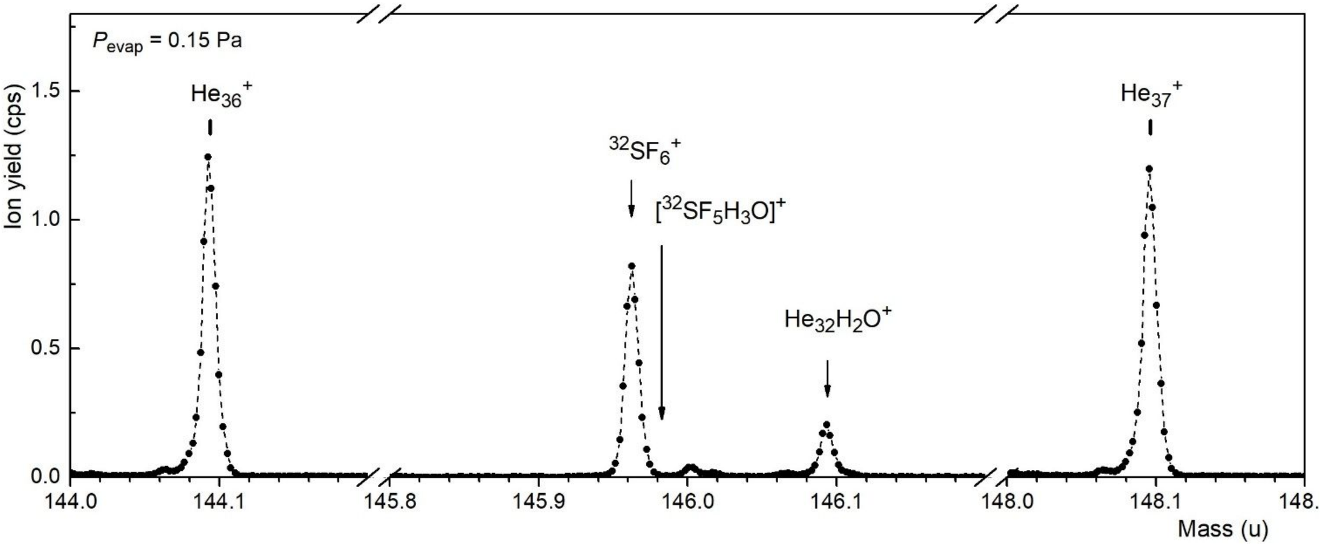

Fig. S1: Three sections of a mass spectrum recorded at an evaporation pressure of 0.15 Pa. Dashed lines connect the data points. The mass peaks due to He_{36}^+ and He_{37}^+ are used to calibrate the mass scale. The expected positions of mass peaks due to $^{32}SF_6^+$ and $He_{32}H_2O^+$ are indicated by arrows; the agreement with the observed peaks is excellent. The hypothetical $[^{32}SF_5H_3O]^+$ ion, on the other hand, is not observed.

S5. Theoretical Results for SF_6^+ and $HeSF_6^+$

Table S1: Cartesian coordinates (in Å) and electronic energies (in Hartree) for atoms and ions optimized at the CCSD/aug-cc-pVDZ level including the ZPE correction.

F
E = -99.547749
F 0.000000 0.000000 0.000000

He
E = -2.889548
He 0.000000 0.000000 0.000000

$HeSF_5^+$
E = -898.308865
S -0.093458 0.033760 -0.000000
F 0.969425 1.193286 -0.000002
F 0.471366 -0.483999 -1.339761
F -1.232499 1.075568 -0.000002
F 0.471364 -0.483993 1.339764
F -1.154707 -1.128040 0.000001
He 2.885385 -1.047780 0.000003

SF_5^+
E = -895.418733
S 0.000000 0.000000 0.000000
F -0.000000 1.543797 0.000000
F 1.336967 -0.771898 -0.000000
F -1.336967 -0.771898 0.000000
F -0.000000 -0.000000 1.572896
F 0.000000 -0.000000 -1.572896

$HeSF_6^+$, IIa
E = -997.859648
S -0.357085 -0.108891 -0.049004
F -0.736331 -1.345081 0.848985
F 0.220626 0.604230 1.192031
F 0.491334 -1.032355 -0.949516
F -1.843592 0.099157 -0.405331
F 0.041243 1.123704 -0.939841
F 2.846466 0.161499 0.148377
He -1.732174 2.620933 0.865854

$HeSF_6^+$, IIb
E = -997.859457
S -0.486684 -0.002489 -0.016635
F 0.453613 1.096170 -0.556346
F -0.032216 -1.074770 0.996739
F -1.949095 -0.027002 -0.507762
F -0.060125 -0.945028 -1.200867
F -0.891831 0.941419 1.176056
F 2.674927 -0.456188 0.323723
He 3.014744 2.114198 -0.908864

$HeSF_6^+$, IIc
E = -997.859370
S 0.443412 -0.011766 0.000003
F 1.218889 -1.381447 -0.000003
F -0.193825 -0.382095 -1.356750
F -0.193802 -0.382092 1.356767
F 1.781230 0.757108 -0.000012
F -0.350785 1.344644 0.000008
F -2.676851 -0.778685 -0.000014
He -1.679143 3.795678 -0.000004

SF_6^+, Ia
E = -994.969542
F -1.462023 -0.382150 -0.000000
S 0.110397 -0.392088 -0.000000
F 0.112237 -1.935227 -0.000000
F -0.755217 2.703288 0.000000
F 0.112237 0.345507 1.356185
F 1.684267 -0.379881 0.000000
F 0.112237 0.345507 -1.356185

SF_6^+, Ib
E = -994.968170
F 0.000000 0.000000 -3.795338
S 0.000000 0.000000 0.558856
F -0.000000 1.336137 1.331833
F -0.000000 -1.336137 1.331833
F 1.573406 -0.000000 0.560905
F -1.573406 0.000000 0.560905
F 0.000000 0.000000 -0.983661

S6. Theoretical Results for $[SF_5H_3O]^+$

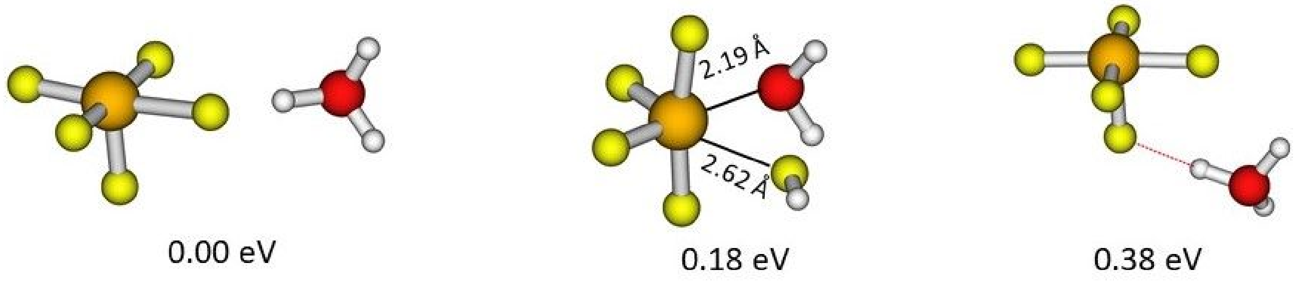

Fig. S2: Three configurations of $[SF_5H_3O]^+$.

Table S2: Dissociation energies of $[SF_5H_3O]^+$, calculated at the B3LYP/aug-cc-pVDZ level (in eV). Calculations at the M06-2X/aug-cc-pVDZ level are provided for comparison.

products	B3LYP	M06-2X
$SF_4^+.H_2O + HF$	0.62	0.65
$H_3O^+ + SF_5$	0.76	0.70
$SF_3H_2O^+.HF + F$	0.95	0.88
$SF_5H^+ + H_2O$	1.34	1.40
$SF_4^+ + H_2O + HF$	1.92	2.09
$SF_5^+.H_2O + H$	3.48	2.83
$SF_5^+ + H_2O + H$	4.88	4.56
$SF_5^+ + H_3O$	5.53	5.33

Table S3: Cartesian coordinates (in Å) and electronic energies (in Hartree) for atoms and ions optimized at the B3LYP and M06-2X levels along with the aug-cc-pVDZ level basis set including the ZPE correction.

B3LYP/aug-cc-pVDZ

F
E = -99.739495
F 0.000000 0.000000 0.000000

H_2O
E = -76.423418
O 0.000000 0.000000 0.117802
H -0.000000 0.764237 -0.471206
H -0.000000 -0.764237 -0.471206

H_3O
E = -76.900955
O 0.000000 0.000000 0.095165
H 0.000000 0.967942 -0.253773
H 0.838263 -0.483971 -0.253773
H -0.838263 -0.483971 -0.253773

H_3O^+
E = -76.680712
O 0.000000 0.000000 0.074828
H 0.000000 0.943588 -0.199541
H 0.817171 -0.471794 -0.199541
H -0.817171 -0.471794 -0.199541

H
E = -0.501657
H 0.000000 0.000000 0.000000

HF
E = -100.451507
F 0.000000 0.000000 0.092564
H 0.000000 0.000000 -0.833073

$SF_4^+.H_2O$
E = -873.568713
S 0.177302 -0.000015 -0.156210
F 1.747119 -0.000216 -0.344327
F 0.075271 -1.646303 -0.269181
F 0.104296 0.000067 1.407615
F 0.075692 1.646328 -0.269289
O -2.008273 0.000094 -0.137621
H -2.396064 -0.790022 -0.561870
H -2.395974 0.790626 -0.561176

$SF_3H_2O^+.HF$
E = -874.268902
S 0.932299 0.318324 -0.166713
F 2.375891 -0.331115 0.081051
F 0.452881 0.117073 1.328031
F 0.323747 -0.983391 -0.820116
O -1.090132 1.090471 -0.582131
F -3.052902 -0.541229 0.292792
H -3.937435 -0.764393 0.102439
H -1.272623 2.025198 -0.396716
H -1.882224 0.570201 -0.317080

SF_4^+
E = -797.097631
S 0.000047 -0.000152 -0.239929
F 0.000086 1.216095 0.729946
F -1.614372 0.001155 -0.515664
F -0.000089 -1.218002 0.727935
F 1.614291 0.001023 -0.515677

$SF_5^+.H_2O$
E = -973.413584
S -0.150741 -0.000243 0.002070
F -0.005132 -1.594482 0.026489
F -1.716670 0.000119 -0.110866
F -0.002299 1.594185 -0.016792
F -0.148498 0.021702 1.591430
O 1.786996 -0.000680 0.162064
H 2.197277 0.804677 -0.217824
H 2.198738 -0.798945 -0.231005
F 0.063696 -0.021125 -1.588127

$SF_5.H_3O^+$
E = -974.043130
S 0.659432 0.019791 -0.178439
F -1.054952 -0.774656 -0.212655
F -0.098528 1.505835 -0.236124
F 1.278140 -1.498130 -0.311354
F 2.110542 0.696360 -0.287893
F 0.613618 -0.029738 1.399253
O -3.289078 0.130008 -0.010972
H -2.294938 -0.236581 -0.103985
H -3.780939 -0.281918 0.724184
H -3.801794 0.064756 -0.838450

$SF_5.H_3O^+$
E = -974.036578
S -0.275005 0.136403 0.000055
F -0.159128 0.070100 1.651620
F -1.786354 -0.287326 0.002685
F -0.164741 0.068640 -1.652008
F -0.455197 1.710436 -0.000392
O 0.236183 -1.993189 0.000856
F 2.337533 0.351546 -0.001360
H 0.774995 -2.218101 0.783868
H 0.762134 -2.222520 -0.789580
H 3.024468 0.983120 -0.006918

$SF_5.H_3O^+$
E = -974.029102
S 0.693926 -0.002458 -0.249467
F 0.670116 1.647503 -0.302481
F -0.338430 0.015195 1.060701
F -0.701969 -0.023206 -1.245860
F 1.985625 0.020611 0.734130
F 0.686893 -1.649825 -0.251261
O -2.921036 -0.076791 0.385486
H -1.970155 -0.006948 0.691703
H -2.962439 0.025921 -0.589325
H -3.522045 0.542190 0.848137

SF_5H^+
E = -897.570390
S -0.241133 -0.000542 -0.142690
F -0.072902 0.015333 1.406169
F -0.152598 1.631764 -0.322689
F -1.798031 -0.057440 -0.285658
F -0.027339 -1.615024 -0.308729
F 2.165414 -0.042324 -0.204945
H 2.827238 0.617906 -0.274307

SF_5
E = -897.334624
S -0.000002 0.000003 -0.203300
F 0.000155 -1.670387 -0.258429
F -1.670491 -0.000089 -0.258522
F 1.670496 0.000222 -0.258523
F -0.000157 1.670429 -0.258251
F 0.000001 -0.000180 1.395146

SF_5^+
E = -896.938902
S 0.000000 0.000000 0.000000
F 0.000000 1.557201 -0.000000
F 1.348576 -0.778600 0.000000
F -1.348576 -0.778600 -0.000000
F 0.000000 0.000000 1.583619
F 0.000000 0.000000 -1.583619

M06-2X/aug-cc-pVDZ

F
E = -99.705436
F 0.000000 0.000000 0.000000

H_2O

E = -76.387120
O -0.000000 0.000000 0.117476
H -0.000000 0.762103 -0.469904
H -0.000000 -0.762103 -0.469904

H_3O
E = -76.856490
O 0.000000 -0.000000 0.103176
H 0.000000 0.950269 -0.275137
H 0.822957 -0.475134 -0.275137
H -0.822957 -0.475134 -0.275137

H_3O^+
E = -76.645514
O 0.000000 -0.000000 0.074808
H 0.000000 0.942332 -0.199488
H 0.816084 -0.471166 -0.199488
H -0.816084 -0.471166 -0.199488

H
E = -0.497898
H 0.000000 0.000000 0.000000

HF
E = -100.413546
F 0.000000 0.000000 0.092136
H 0.000000 0.000000 -0.829224

$SF_4^+.H_2O$
E = -873.366712
S 0.165540 -0.000020 -0.167246
F 1.718503 -0.000336 -0.313099
F 0.059043 -1.614571 -0.263625
F 0.082662 0.000013 1.376702
F 0.059759 1.614613 -0.263581
O -1.909852 0.000344 -0.138248
H -2.324936 -0.794389 -0.522027
H -2.324587 0.794480 -0.523631

$SF_3H_2O^+.HF$
E = -874.066607
S 0.870500 0.367763 -0.037748
F 2.183082 -0.509698 -0.008079
F 0.276436 -0.296564 1.243909
F 0.181889 -0.513130 -1.122210
O -1.019301 1.383850 -0.165583
F -2.572860 -0.769948 0.074362
H -3.405390 -1.171128 -0.007660
H -1.169566 2.199646 0.334632
H -1.815562 0.820534 -0.090194

SF_4^+
E = -796.926912
S -0.000017 -0.000033 -0.244731
F 0.000118 1.202429 0.707925
F -1.585615 -0.000014 -0.490363
F 0.000084 -1.202315 0.708027
F 1.585443 -0.000040 -0.490511

$SF_5^+.H_2O$
E = -973.202222
S -0.139557 -0.000056 0.001128
F 0.000147 -1.571653 0.034541
F -1.683753 -0.002304 -0.128966
F -0.000004 1.571880 -0.023155
F -0.165066 0.029073 1.568847
O 1.726121 0.002259 0.170740
H 2.153920 0.806713 -0.190198
H 2.153848 -0.806312 -0.180960
F 0.083806 -0.028949 -1.563801

$SF_5.H_3O^+$
E = -973.804213
S 0.592506 0.015947 -0.186166
F -0.854260 -1.030111 -0.135406
F -0.466089 1.296534 -0.165371
F 1.493820 -1.302501 -0.331681
F 1.836109 0.987409 -0.322906
F 0.627541 -0.025265 1.373480
O -3.042856 0.040731 -0.142375
H -2.100777 -0.413497 -0.113970
H -3.027482 0.966143 0.163306
H -3.743088 -0.468227 0.305280

$SF_5.H_3O^+$
E = -973.798769
S -0.152314 0.192968 0.000006
F -0.097198 0.063733 1.615925
F -1.661584 0.569620 0.004282
F -0.104327 0.070184 -1.616442
F 0.421071 1.644240 0.001810
O -0.687680 -1.827152 -0.003060
F 2.232244 -0.554662 -0.002061
H -1.140437 -2.151283 0.795412
H -1.161739 -2.145310 -0.791523
H 3.128793 -0.311719 -0.011133

$SF_5.H_3O^+$
E = -973.794227
S 0.676688 0.001592 0.250824
F 0.627996 -1.617010 0.303179
F -0.264829 -0.012155 -1.079010
F -0.745072 0.036064 1.156783
F 1.995627 -0.035481 -0.645899
F 0.674257 1.617973 0.243634
O -2.887159 0.078712 -0.363525

H -1.979630 0.011340 -0.759535
H -2.813085 -0.029012 0.609268
H -3.528840 -0.542011 -0.762907

SF_5H^+
E = -897.365635
S -0.251118 -0.000004 -0.151260
F -0.020286 0.015993 1.366556
F -0.157033 1.597495 -0.320875
F -1.788415 -0.048336 -0.234611
F -0.051622 -1.584221 -0.307160
F 2.141216 -0.036191 -0.205555
H 2.903161 0.497406 -0.265038

SF_5
E = -897.133095
S 0.000045 0.000015 -0.209526
F -1.636571 -0.008520 -0.248436
F -0.009127 1.636416 -0.248936
F 0.007930 -1.636415 -0.248979
F 1.636873 0.008533 -0.249244
F 0.000816 -0.000040 1.368086

SF_5^+
E = -896.751698
S 0.000000 0.000000 0.000000
F 0.000000 1.534091 -0.000000
F 1.328562 -0.767046 0.000000
F -1.328562 -0.767046 0.000000
F 0.000000 0.000000 1.562457
F 0.000000 0.000000 -1.562457

S7. References

(1) Laimer, F.; Kranabetter, L.; Tiefenthaler, L.; Albertini, S.; Zappa, F.; Ellis, A. M.; Gatchell, M.; Scheier, P. Highly Charged Droplets of Superfluid Helium. *Phys. Rev. Lett.* **2019,** *123*, 165301.

(2) Tiefenthaler, L.; Kollotzek, S.; Gatchell, M.; Hansen, K.; Scheier, P.; Echt, O. Isotope Enrichment in Neon Clusters Grown in Helium Nanodroplets. *J. Chem. Phys.* **2020,** *153*, 164305.

(3) Mauracher, A.; Echt, O.; Ellis, A. M.; Yang, S.; Bohme, D. K.; Postler, J.; Kaiser, A.; Denifl, S.; Scheier, P. Cold Physics and Chemistry: Collisions, Ionization and Reactions inside Helium Nanodroplets Close to Zero K. *Phys. Rep.* **2018,** *751*, 1-90.

(4) Patterson, P. L. Evidence of the Existence of an He_3^+ Ion *J. Chem. Phys.* **1968,** *48*, 3625-3631.

(5) Evans, M.; Ng, C. Y.; Hsu, C. W.; Heimann, P. A High Resolution Energy-Selected Kinetic Energy Release Study of the Process $SF_6 + h\nu \rightarrow SF_5^+ + F + e^-$: Heat of Formation of SF_5^+. *J. Chem. Phys.* **1997,** *106*, 978-981.
